# The Probable Association between Chronic *Toxoplasma gondii* Infection and Type 1 and Type 2 Diabetes Mellitus: A Case-Control Study

**DOI:** 10.1155/2021/2508780

**Published:** 2021-05-24

**Authors:** Shahrzad Soltani, Sanaz Tavakoli, Mohamad Sabaghan, Mehdi Sagha Kahvaz, Marzieh Pashmforosh, Masoud Foroutan

**Affiliations:** ^1^USERN Office, Abadan Faculty of Medical Sciences, Abadan, Iran; ^2^Department of Parasitology and Mycology, School of Medicine, Isfahan University of Medical Sciences, Isfahan, Iran; ^3^Behbahan Faculty of Medical Sciences, Behbahan, Iran

## Abstract

**Purpose:**

The probable association between *Toxoplasma gondii* (*T. gondii*) infection and diabetes mellitus (DM) is still controversial, and there are several studies with conflicting results. Thus, this study was performed to assess the possible association between chronic *T. gondii* infection and type 1 diabetes mellitus (T1DM) and T2DM.

**Methods:**

In this case-control study, a total of 105 diabetic subjects including 36 patients with T1DM and 69 patients with T2DM were recruited. In addition, 150 nondiabetic subjects were enrolled as controls. Each case group had its own control group. Each participant completed a structured questionnaire obtaining demographic information. Serum samples were examined for *T. gondii-*specific IgG antibody using enzyme-linked immunosorbent assay (ELISA) method.

**Results:**

Analysis revealed that 69.4% and 34.0% of patients with T1DM and control subjects were serologically positive for *T. gondii,* respectively (odds ratio (OR): 4.41; 95% confidence interval (CI): 1.75–11.06; *P*=0.001). Moreover, 72.5% of T2DM patients and 29.0% of healthy individuals were seropositive for *T. gondii* (OR: 6.44; 95% CI: 3.25–12.74; *P* < 0.001). Among risk factors, only contact with cats was significantly associated with IgG seroprevalence in both T2DM patients (*P* < 0.001) and control subjects (*P*=0.045).

**Conclusion:**

Although the results showed that chronic *T. gondii* infection is significantly associated with T1DM and T2DM, there remain many questions regarding the exact mechanisms of *T. gondii* in the pathogenesis of DM.

## 1. Introduction

*Toxoplasma gondii* (*T. gondii*) is an obligate apicomplexan intracellular parasite that is capable of infecting nearly all warm-blooded animal species, including humans [1]. There are various routes of *T. gondii* transmission to humans: ingestion of oocyst-contaminated food or water, eating cyst-infected raw meat, vertical transmission from mother to fetus, organ transplantation, and blood transfusion [2–5]. It is estimated that one-third of the human population worldwide are infected with this parasite [2, 6, 7]. Previous systematic review articles in Iran have reported high *T. gondii* seroprevalence rates of more than 45% in various human groups, including HIV/AIDS patients, cancer patients, transplant recipients, and hemodialysis patients when compared to lower seroprevalence rates observed in the general population including healthy blood donors and pregnant women [8, 9].

Diabetes mellitus (DM) is one of the major worldwide public health concerns of the 21st century. It is estimated that the number of persons suffering from DM will increase to 552 million (7.7%) in 2030 [10]. Diabetic patients have suppressed immune systems, potentially indicating that these subjects may be more susceptible to acquire *T. gondii* [11–13]. Type 1 diabetes mellitus (T1DM) is characterized by hyperglycemia due to the deficiencies in insulin hormone release, while type 2 diabetes mellitus (T2DM) is hallmarked by the failure to properly respond to insulin [10].

Since previous studies on the possible association between *T. gondii* infection and DM have reported conflicting results [11, 13], we decided to conduct a case-control investigation to shed light on the probable association between chronic *T. gondii* infection and T1DM and T2DM.

## 2. Materials and Methods

### 2.1. Study Area

**Figure 1:**
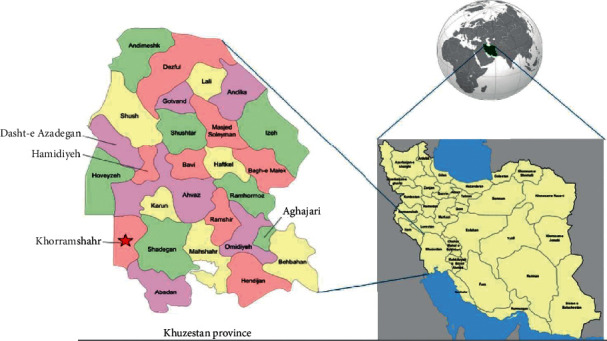
Location of Khorramshahr city. The study region is shown with red asterisk.

The study was carried out in Khorramshahr city (Khuzestan province, southwest Iran, 30.4256°N, 48.1891°E) (Figure 1). At the 2016 census, its population was 170,976. Khorramshahr city has hot summers (up to 55°C) and cold winters (1°C). The annual rainfall is around 140 mm.

### 2.2. Study Design and Sample Collection

In this case-control study, a total of 105 cases including 36 patients with T1DM and 69 patients with T2DM were recruited from Valiasr Hospital (affiliated to the Abadan Faculty of Medical Sciences) from December 2019 to March 2020. A total of 150 control subjects were also enrolled. Each case group had its own control group. In the diabetic groups, inclusion criteria were as follows: fasting plasma glucose greater than or equal to 7.0 mmol/L and/or 2-hour plasma glucose greater than or equal to 11.1 mmol/L [14]. Healthy individuals were defined as control group if they had no previous history of diagnosis of diabetes and had fasting and 2-hour glucose measures under the common thresholds for diabetes. The diabetic patients in both case groups with metabolic disorders and those receiving immunosuppressive drugs were excluded from the current research.

### 2.3. Questionnaire

Each participant completed a structured questionnaire which obtained the following demographic information: age, gender, residence, education level, contact with cat, source of drinking water, and consumption of raw or undercooked meat.

### 2.4. Serological Assay

All the patients and control subjects had 5 mL of venous blood drawn. The samples were then centrifuged at 1700 ×g for 5 minutes and kept at −20°C till tested. In order to detect anti-*T. gondii* IgG antibody titer in the sera, a commercially available (Torch-IgG, Trinity Biotech Company) enzyme-linked immunosorbent assay (ELISA) kit was used according to the manufacturer's instructions.

### 2.5. Statistical Analysis

All data were imported into the Statistical Package for the Social Sciences (SPSS) software (version 21) (SPSS Inc., Chicago, IL, USA) for analysis. Chi-square and Fisher's exact tests were used to compare the variables. The significance level was defined to be less than 0.05 (*P* < 0.05).

## 3. Results

### 3.1. Seroepidemiology of *T. gondii* Infection in T1DM Patients

The seroprevalence of *T. gondii* infection in T1DM and control subjects was estimated to be 69.4% (25/36) and 34.0% (17/50), respectively, which showed a statistically significant difference (odds ratio (OR): 4.41; 95% confidence interval (CI): 1.75–11.06; *P*=0.001). Demographic characteristics of patients with T1DM and nondiabetic subjects, such as age group, gender, residence, education level, source of drinking water, and consumption of raw/undercooked meat, are presented in Table 1. T1DM patients in the age group of 21–30 years (80.0%) showed the highest seroprevalence. No significant difference was observed between females (82.35%) and males (57.89%) of T1DM patient group (*P*=0.109). T1DM patients who lived in rural areas (81.81%) had higher seroprevalence of *T. gondii* than those who were in urban regions (64.0%), but no statistically significant difference was observed (*P*=0.254). In addition, the seroprevalence of *T. gondii* infection in T1DM patients with different education levels was not significantly different (*P*=0.261). *T. gondii* seroprevalence was not significantly different among T1DM patients with the history of contact with cats (*P*=0.073), source of drinking water (*P*=0.571), and consumption of raw/undercooked meat (*P*=0.609) (Table 1).

### 3.2. Seroepidemiology of *T. gondii* Infection in T2DM Patients

T2DM patients (72.5%) showed a higher seroprevalence of *T. gondii* infection than nondiabetic group (29.0%) (OR: 6.44; 95% CI: 3.25–12.74; *P* < 0.001). T1DM patients in the age group of more than 60 years showed the highest rate of infection with *T. gondii* (73.91%). No significant difference was observed between males (67.64%) and females (77.14%) in T2DM patients (*P*=0.377). About 74.41% of T2DM patients living in urban areas were seropositive for *T. gondii*, while in rural regions 69.23% were found to be IgG-positive (*P*=0.64). The seroprevalence of *T. gondii* infection was not significantly different in T2DM patients with different educational levels (*P*=0.21)*. T. gondii* seroprevalence was not significantly different among T1DM patients and source of drinking water (*P*=0.292) and consumption of raw/undercooked meat (*P*=0.384). Among risk factors, only contact with cats was significantly associated with IgG seroprevalence in both T2DM patients (*P* < 0.001) and control subjects (*P*=0.045) (Table 2).

## 4. Discussion

The possible association between toxoplasmosis and DM is still controversial, as there are several studies with conflicting results [11, 13, 15–17]. Since there is a lack of knowledge about the epidemiological status of *T. gondii* infection and its association with T1DM and T2DM in southwest Iran (Khuzestan province, Khorramshahr city), anti-*T. gondii* IgG antibody in diabetic patients compared to nondiabetic subjects was evaluated. Our findings showed higher seroprevalence of anti-*T. gondii* IgG antibody in T1DM and T2DM patients in comparison to nondiabetic individuals. Thus, the results of our study based on ELISA method supported the association between chronic toxoplasmosis and both types of DM.

T1DM is considered as an autoimmune disease, which is probably associated with genetic and environmental factors [10]. The association between infectious agents and T1DM has been approved [18, 19]. In this study, higher seroprevalence of *T. gondii* infection in T1DM patients in comparison to nondiabetic individuals was observed (69.4% versus 34.0%). *T. gondii* can infect all nucleated cells, including pancreatic *β*-cells. Pancreas can secret insulin, which is crucial for controlling blood glucose level. Any deficiency in insulin production may cause the occurrence of T1DM. Therefore, *T. gondii* infection could develop T1DM [12, 16, 20]. In the other hand, the diabetic patients are considered as immunocompromised subjects and are more vulnerable to infection with *T. gondii* than healthy individuals [21].

T2DM is a metabolic disease and, as a major global health concern, its incidence rate has increased during the recent decade throughout the globe [10, 22]. In the current study, 72.5% of T2DM patients and 29.0% of nondiabetic subjects were seropositive for anti-*T. gondii* IgG antibody, and the difference was statistically significant (*P* < 0.001). The same results were reported by Ozcelik et al. from Turkey [23]. In contrast with the results of our study, Molan et al. reported that 62.0% and 66.0% of the T2DM patients and nondiabetic subjects were seropositive for *T.gondii* infection, respectively, but the difference was not statistically significant [17]. In a review paper with meta-analysis approach, Majidiani et al. reviewed seven articles to investigate the association between *T. gondii* infection and DM from a global perspective. They concluded that latent toxoplasmosis accounts as a possible risk factor for T2DM (OR: 2.39; 95% CI: 1.20–4.75; *P*=0.013), while no statistically significant association was observed between *T. gondii* and T1DM (OR: 1.10; 95% CI: 0.13–9.57; *P*=0.929) [13]. The discordance between studies could be explained due to the study area, the number of participants in the case and control groups, different type of sampling, environmental factors, lifestyle and habits of the people as well as different specificity and sensitivity of the laboratory techniques, variable cutoff values, or antibody titers for serological kits.

In the current research, the main risk factors of *T. gondii* infection were assessed. A significant association between *T. gondii* seroprevalence and contact with cats was found in both T2DM patients and nondiabetic subjects. In the previous studies among general population and patients undergoing hemodialysis, the same results were observed in southwest Iran [24, 25]. Since cats are considered as the only definitive hosts and are one of the major sources of *T. gondii*, it seems that close contact with cats is considered as an important risk factor for acquiring the infection. The cats can release several millions of oocysts into the environment and public places through feces [1, 26]. In addition, the sporulated oocysts have the ability to survive for a long time in the optimum conditions in the soil [5]. Based on a review paper, the prevalence of *T. gondii* oocysts was estimated to be 16% (95% CI: 10–26) in the soil of public places worldwide [5].

Choosing the appropriate inclusion and exclusion criteria, investigating the clinical and diagnostic history of all the participants, assessment of demographic information and the main risk factors of *T. gondii* infection through a structured questionnaire, and investigation of both T1DM and T2DM are strengths of the current study. Nonetheless, there are limitations that should be kept in mind: (1) this study was based on sampling from a small number of T1DM and T2DM patients in a limited area; (2) only serological assay by ELISA was performed on samples with no supporting data by molecular confirmation.

## 5. Conclusion

In conclusion, we found high rates of *T. gondii* seroprevalence in diabetic patients in southwest Iran. Although this study revealed a significant association between chronic *T. gondii* infection and two types of diabetes mellitus (T1DM and T2DM), there remain many questions regarding the exact mechanisms of *T. gondii* in the pathogenesis of DM. More studies are required to elucidate the exact association between *T. gondii* and DM.

## Figures and Tables

**Table 1:** Demographic characteristics and risk factors related to the seroprevalence of *T. gondii* infection in T1DM patients, Khorramshahr city.

Characteristic	Type 1 DM (*N* = 36)				Controls (*N* = 50)				Type 1 DM *versus* controls
	No. of tested	IgG-positive	%	*P* value	No. of tested	IgG-positive	%	*P* value	*P* value
Age									
0–10	5	3	60.00	0.847	8	2	25.00	0.911	0.249
11–20	9	6	66.66		12	4	33.33		0.142
21–30	10	8	80.00		15	5	33.33		**0.029**
31–40	12	8	66.66		15	6	40.00		0.161

Gender									
Female	17	14	82.35	0.109	25	9	36.00	0.765	**0.003**
Male	19	11	57.89		25	8	32.00		0.086

Residence									
Urban	25	16	64.00	0.254	30	10	33.33	0.903	**0.023**
Rural	11	9	81.81		20	7	35.00		**0.016**

Education level									
Diploma or lower	27	20	74.07	0.261	35	12	34.28	0.948	**0.002**
University degree	9	5	55.55		15	5	33.33		0.26

Contact with cat									
Yes	24	19	79.16	0.073	36	13	36.11	0.438	**0.002**
No	12	6	50.00		14	4	28.57		0.237

Source of drinking water									
Unpurified water	9	6	66.66	0.571	9	4	44.44	0.358	0.319
Purified water	27	19	70.37		41	13	31.70		**0.002**

Consumption of raw/undercooked meat									
Yes	6	4	66.66	0.609	10	5	50.00	0.204	0.451
No	30	21	70.00		40	12	30.00		**0.001**

Total	36	25	69.4		50	17	34.0		**0.001**

**Table 2:** Demographic characteristics and risk factors related to the seroprevalence of *T. gondii* infection in T2DM patients, Khorramshahr city.

Characteristic	Type 2 DM (*N* = 69)				Controls (*N* = 100)				Type 2 DM *versus* controls
	No. of tested	IgG-positive	%	*P* value	No. of tested	IgG-positive	%	*P* value	*P* value
Age									
≤40	12	8	66.66	0.884	25	7	28.00	0.93	**0.03**
41–60	34	25	73.52		50	14	28.00		**<0.001**
>60	23	17	73.91		25	8	32.00		**0.004**

Gender									
Female	35	27	77.14	0.377	50	13	26.00	0.509	**<0.001**
Male	34	23	67.64		50	16	32.00		**0.001**

Residence									
Urban	43	32	74.41	0.64	60	19	31.66	0.472	**<0.001**
Rural	26	18	69.23		40	10	25.00		**<0.001**

Education level									
Diploma or lower	51	39	76.47	0.21	66	21	31.81	0.387	**<0.001**
University degree	18	11	61.11		34	8	23.52		**0.007**

Contact with cat									
Yes	52	44	84.51	**<0.001**	73	25	34.24	**0.045**	**<0.001**
No	17	6	35.29		27	4	14.81		0.114

Source of drinking water									
Unpurified water	7	4	57.14	0.292	11	6	54.54	**0.048**	0.648
Purified water	62	46	74.19		89	23	25.84		**<0.001**

Consumption of raw/undercooked meat									
Yes	8	5	62.50	0.384	14	7	50.00	0.062	0.454
No	61	45	73.77		86	22	25.58		**<0.001**

Total	69	50	72.5		100	29	29.00		**<0.001**

## Data Availability

The datasets used and/or analysed during the current study are available from the corresponding author upon reasonable request.

## Abbreviations

CI: Confidence interval
DM: Diabetes mellitus
ELISA: Enzyme-linked immunosorbent assay
IgG: Immunoglobulin G
OR: Odds ratio
*T. gondii*: *Toxoplasma gondii*
T1DM: Type 1 diabetes mellitus
T2DM: Type 2 diabetes mellitus.
